# Comparison of two commercial recirculated aquacultural systems and their microbial potential in plant disease suppression

**DOI:** 10.1186/s12866-021-02273-4

**Published:** 2021-07-05

**Authors:** Sammar Khalil, Preeti Panda, Farideh Ghadamgahi, AnnaKarin Rosberg, Ramesh R Vetukuri

**Affiliations:** 1 Department of Biosystems and Technology, Swedish University of Agricultural Sciences, Box 103, 230 53 Alnarp, Sweden; 2 Plant and Food Research, Plant Protection, Lincoln, New Zealand; 3 Department of Plant Breeding, Swedish University of Agricultural Sciences, Box 102, 230 53 Alnarp, Sweden

**Keywords:** Aquaponics, Tilapia, Clarias, *Pseudomonas flourescens*, *Pseudomonas veronii*, plant growth promotion, *in vitro* antagonistic

## Abstract

**Background:**

Aquaponics are food production systems advocated for food security and health. Their sustainability from a nutritional and plant health perspective is, however, a significant challenge. Recirculated aquaculture systems (RAS) form a major part of aquaponic systems, but knowledge about their microbial potential to benefit plant growth and plant health is limited. The current study tested if the diversity and function of microbial communities in two commercial RAS were specific to the fish species used (Tilapia or Clarias) and sampling site (fish tanks and wastewaters), and whether they confer benefits to plants and have *in vitro* antagonistic potential towards plant pathogens.

**Results:**

Microbial diversity and composition was found to be dependent on fish species and sample site. The Tilapia RAS hosted higher bacterial diversity than the Clarias RAS; but the later hosted higher fungal diversity. Both Tilapia and Clarias RAS hosted bacterial and fungal communities that promoted plant growth, inhibited plant pathogens and encouraged biodegradation. The production of extracellular enzymes, related to nutrient availability and pathogen control, by bacterial strains isolated from the Tilapia and Clarias systems, makes them a promising tool in aquaponics and in their system design.

**Conclusions:**

This study explored the microbial diversity and potential of the commercial RAS with either Tilapia or Clarias as a tool to benefit the aquaponic system with respect to plant growth promotion and control of plant diseases.

## Background

Major challenges such as climate changes, population increases, limited availability of natural resources, and pandemics threaten food security [1], raising urgent needs to shift to robust and sustainable food production systems [2, 3]. As one of the largest food industries globally for animal protein production, aquaculture could play a major role in meeting these needs. Its future expansion will largely rely on land-based recirculated aquaculture systems (RAS), which enable better control of rearing conditions with significantly lower water consumption and release of nutrients (organic matter, nitrogen and phosphorous) into lakes, rivers and the sea [4]. However, accumulation of nitrates (which is harmful to fish) under RAS conditions is problematic [5]. This can be ameliorated by plant uptake, so a potential solution is to integrate RAS and hydroponic systems for plant cultivation in ‘aquaponic systems’. Thus, aquaponic systems are promising future food production systems with robust environmental profiles and potential to enhance food security. However, their sustainability needs further improvement, as they are complex and more knowledge is needed concerning ideal plant nutrient balances in relation to amounts and types of fish feed, system design, and resilience towards pathogen attack and spread of diseases [6]. These aspects are strongly related to RAS conditions. For example, the lack or low availability of elements in fish feed required for plant growth - such as phosphorous and iron - frequently limits aquaponic systems’ productive efficiency [7]. Hence, these nutrients are currently maintained at required levels by adding extra phosphorus and iron to the systems. Stabilization of the RAS element of aquaponic systems, in terms of water quality parameters such as temperature and pH, is also crucial to meet fish, plant and microbial requirements optimally [8, 9] and thereby promote good plant and fish growth.

Nevertheless, sustainable approaches for controlling fish, human and plant pathogens in aquaponic systems are also needed [6]. Plant root diseases caused by fungal pathogens such as *Fusarium*, *Verticllium*, oomycetes such as *Pythium* and *Phytophthora* spp., or bacterial pathogens such as *Ralstonia* and *Xanthomonas* spp. are commonly found in aquatic environments including hydroponic systems and hence aquaponic systems [10–13]. Biotic and abiotic means to control these pathogens in hydroponic systems, including exploitation of natural microbial communities’ suppressive potential, have been investigated [14–19]. However, further study of the suppressive potential of natural microbial communities in aquaponic systems is needed. This is due to the complexity of the systems and associated variables related to water quality, fish feed, the fish, plants and microbial taxa present in compartments from the biofilter to the hydroponic unit (in and through which pathogens may enter and excessively grow if not controlled). Restrictions governing pesticides and antibiotics to control plant and fish diseases, respectively, highlight the need to provide solutions that enhance the sustainability of aquaponic systems towards pathogen attack.

Microbes can suppress pathogens in various ways, including competition, production of antibiotics and extracellular enzymes, and induction of plant resistance or growth-promotion [20]. Microbial communities also have confirmed roles in nutrient recycling [21], plant growth promotion [22], and protection against pathogen attack [23] in aquaponic systems. Hence, the potential utility of modulating the microbial habitat and community in the RAS component of aquaponic systems to counter fish diseases has been addressed [24].

However, more research into microbes’ roles and activities in RAS is needed to optimize their promotion of plant growth and suppression of plant diseases. Thus, the objective of the study presented here was to elucidate microbial diversity in a commercial RAS (with no aquaponic connection) and its potential to promote plant growth and act against plant pathogens. For these purposes, variations in the microbial community between systems with two fish species, Tilapia (*Oreochromis niloticus*) or Clarias (*Clarias gariepinus*), and between two sampling sites: the water tank with fish biosolids and wastewater have been examined in the current study. The communities’ functional roles in terms of production of extracellular enzymes with known activities against plant pathogens and in nutrient solubilization were also examined. The study is based on the following hypotheses. First, a RAS (with no aquaponic connection) hosts microbial communities that are beneficial to plants and antagonistic to pathogens, with characteristics that depend on the fish species used and sampling site in the system. Second, the production of extracellular enzymes and antagonistic potential to control plant pathogens *in vitro* are fish species- and site-specific.

## Results

### RAS conditions

Conditions at the sample collection time in the two types of RAS, recirculated aquaculture system, with different fish species were similar in terms of water quality parameters such as pH, temperature, conductivity and contents of both ammonium and nitrate (Table 1). However, the total weight of Tilapia per tank was far lower than the corresponding weight of Clarias (ca. 50 and 160 kg, respectively, at the sampling time). Both species were fed with the same commercial feed, supplied by Skrettting (https://www.skretting.com/en/), but with a slight difference in composition and larger differences in daily amounts. Tilapia were fed five times per day and Clarias 18 times per day.

**Table 1:** Growth conditions in each recirculated aquaculture system (RAS) populated with either Tilapia or Clarias

Cultivation factors	RAS with Tilapia	RAS with Clarias
Temperature	21 ^0^ C	20.9 ^0^ C
pH	7.7	8.5
Conductivity**	130 mS cm^-1^	180 mS cm^-1^
Ammonium content	2.1 mg / liter	1.8 mg / liter
Nitrate content	280 mg / liter	188 mg / liter
Nitrite content	0,59 mg / liter	0,18 mg / liter
Fish weight	40-600 g per tank	100-4000 g per tank
Feed composition	37 % protein + 10 % fat	44 % protein + 12 % fat
Daily amount	5 times	18 times

** Electrical conductivity

### Microbial abundance

**Fig. 1:**
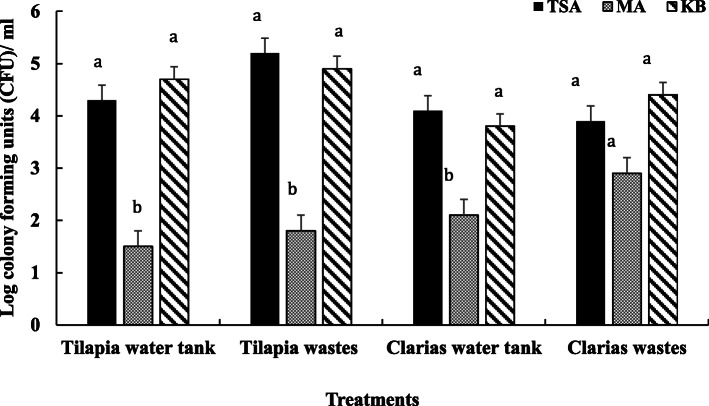
Microbial colonies isolated from water and waste samples from Tilapia and Clarias RAS and enumerated on 0.1 % Tryptic soya agar (TSA) complemented with cycloheximide (100 µL mL^− 1^) for enumeration of the general bacterial flora; 0.5 % malt extract agar (MA) for enumeration of the general fungal flora; and on King Agar B (KB) with cycloheximide (100 µg mL^− 1^) for enumeration of fluorescent pseudomonads. Letters above the bars indicate the significant differences between the treatments

Microbial enumeration on selective media indicated more general bacterial flora and *Pseudomonas fluorescens* in samples collected from the Tilapia RAS system than the Clarias RAS system (Fig. 1). However, there were differences in amounts of general fungal flora, which were more abundant in samples of Clarias wastewater than in Clarias water tank samples or either type of Tilapia RAS samples.

### Diversity of the microbial communities

**Fig. 2:**
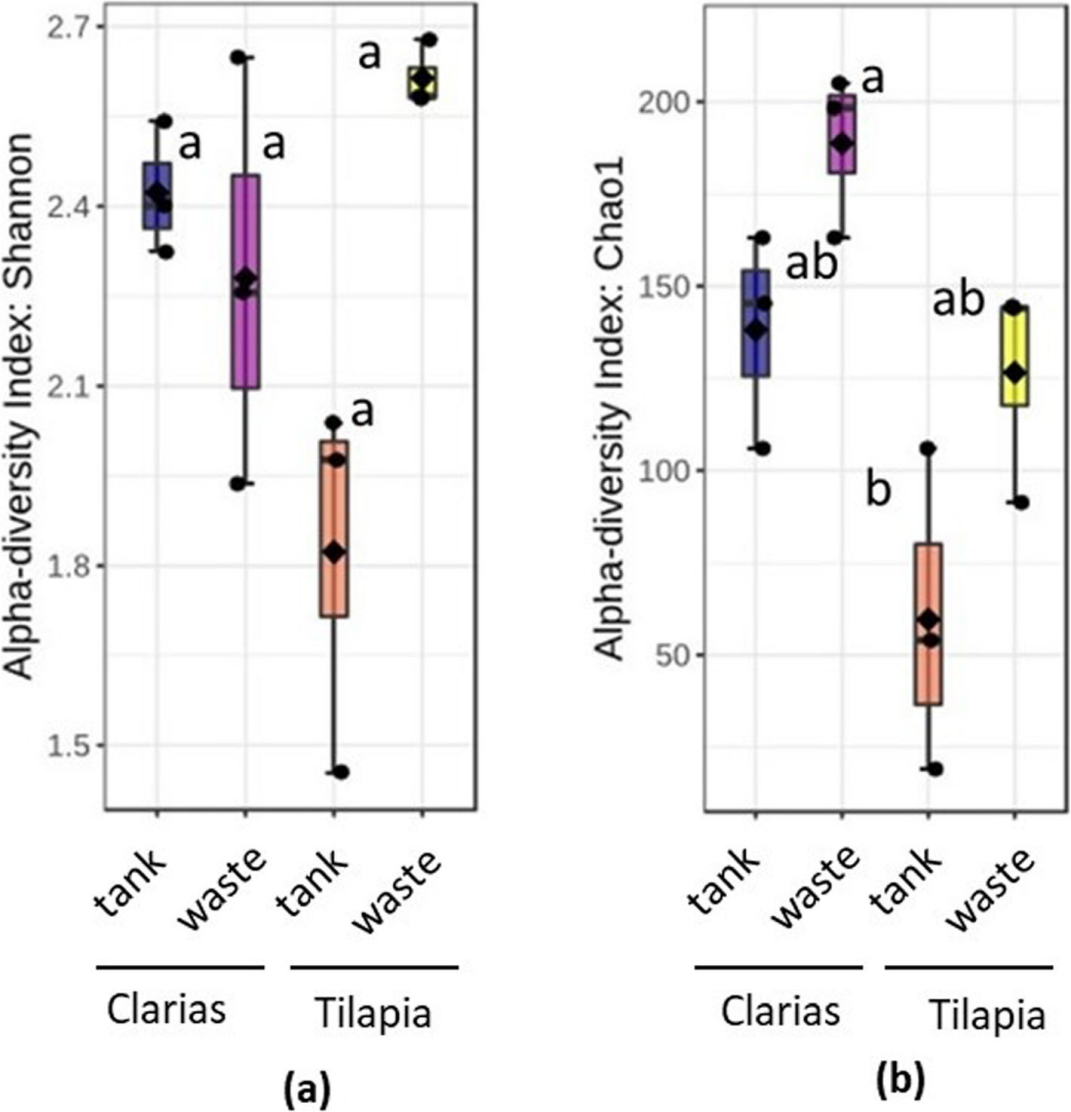
Alpha diversity of bacterial communities in the fish water tank and the waste water sampling sites of the Tilapia and Clarias RAS as judged by the diversity indices (**a**) Shannon and (**b**) Chao1. Letters above the boxplots indicate the significant differences between the treatments

Processing Ilumina MiSeq sequencing data revealed the presence of 4,558 bacteria operational taxonomic units (OTUs) and 405 fungal OTUs in samples of the two RAS. Total read counts for the bacterial and fungal datasets were 647, 232 and 530,351, respectively. The bacterial communities had significantly higher alpha diversity (according to Shannon indices) in Tilapia RAS water tank samples (p = 0.021) than in Clarias RAS water tank samples (Fig. 2a). However, bacterial alpha diversity did not significantly differ (p < 0.05) between Tilapia RAS samples (water or waste) and Clarias RAS wastewater samples. Calculated Chao1 indices also indicated that alpha diversity was significantly higher (p = 0.012) in samples of Tilapia RAS wastewater than in Clarias RAS water tank samples, which had the lowest Chao1 indices (Fig. 2b). In further accordance with the Shannon indices, Chao1 indices did not significantly differ between Tilapia RAS samples (tank or wastewater) and Clarias RAS wastewater samples.

**Fig. 3:**
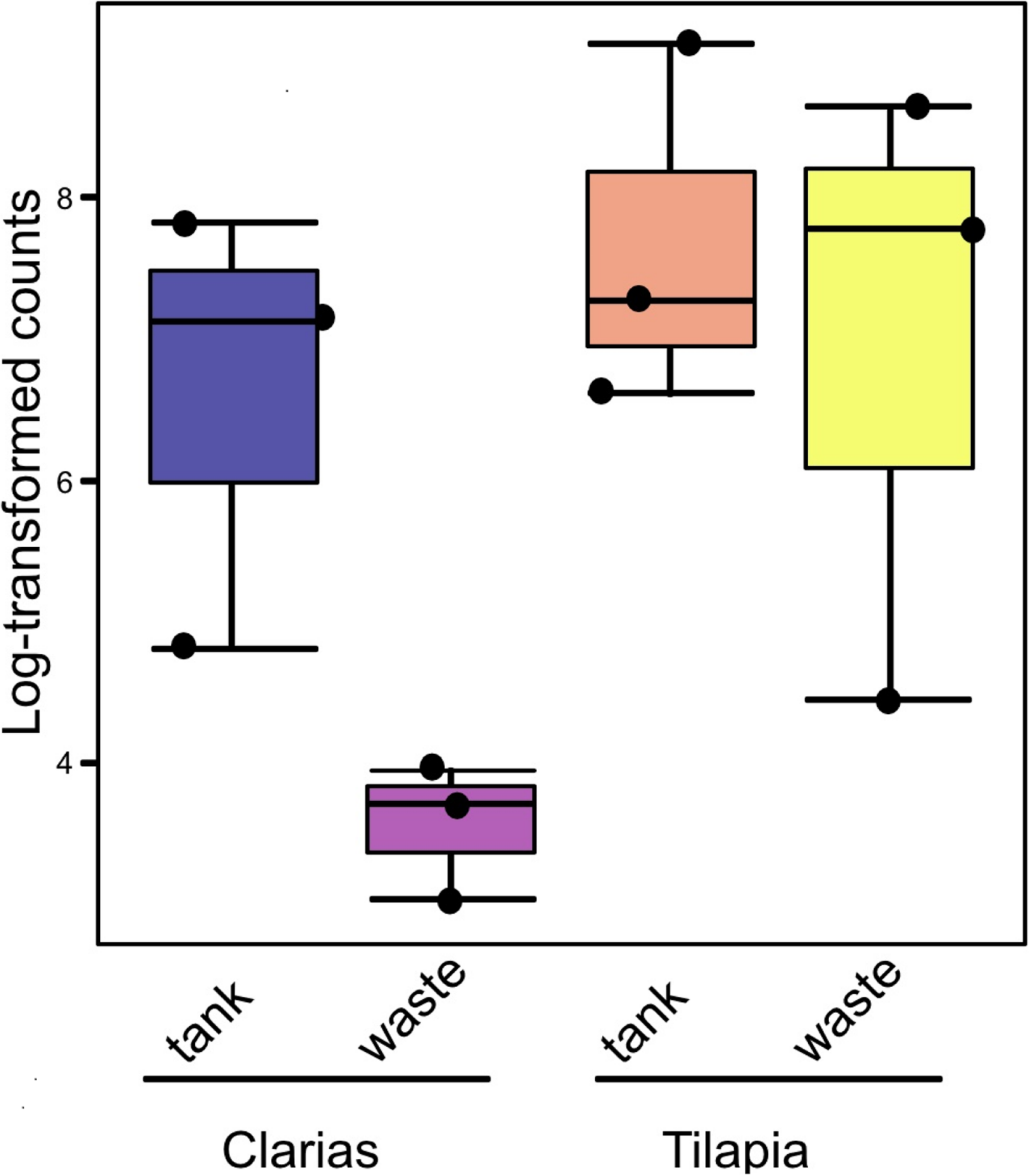
Log-transformed counts of *Pseudomonas* communities in the water tank and wastewater site from the Tilapia and Clarias RAS. Letters above the boxplots indicate the significant differences between the treatments

**Fig. 4:**
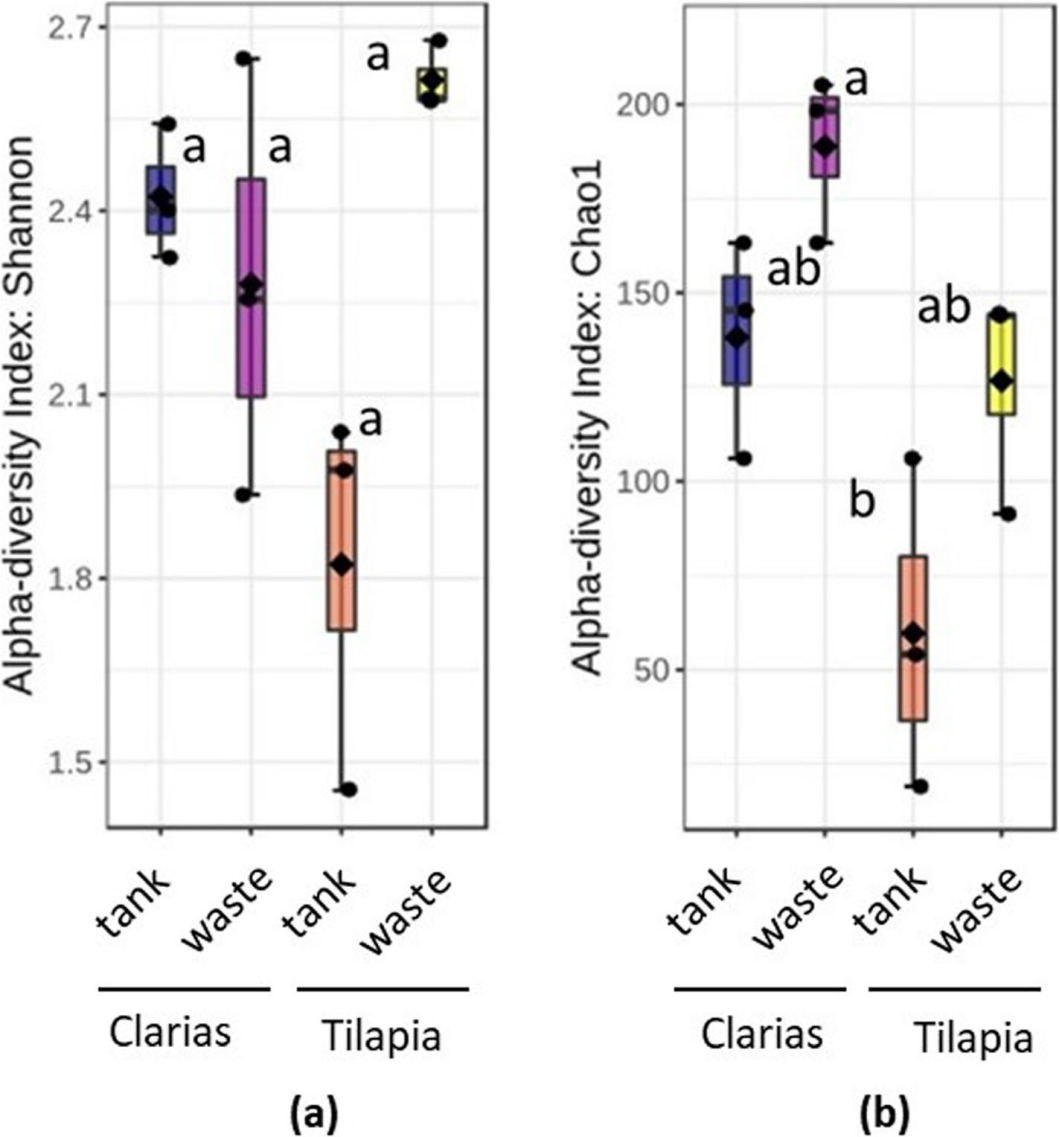
Alpha diversity of fungal communities from the water tank and the fish waste sample sites in the recirculated aquaculture systems populated with two different fish species -Tilapia or Clarias, as judged by the diversity indices (**a**) Shannon and (**b**) Chao1. Letters above the boxplots indicate the significant differences between the treatments

Regarding abundance of *Pseudomonas*, no significant differences (p < 0.05) in log-transformed *Pseudomonas* counts between the four types of samples (Tilapia and Clarias RAS tank water and wastewater) were found, as shown in Fig. 3. However, although we found no significant differences between treatments in fungal community alpha diversity in terms of Shannon indices (p = 0.211) (Fig. 4a), there were highly significant differences in Chao1 indices (p = 0.008) between treatments. More specifically, they clearly indicated that fungal diversity was higher in the Clarias RAS wastewater than in the Tilapia RAS water tank, although Chao 1 indices did not significantly differ between any other pairs of sample types (Fig. 4b).

**Fig. 5:**
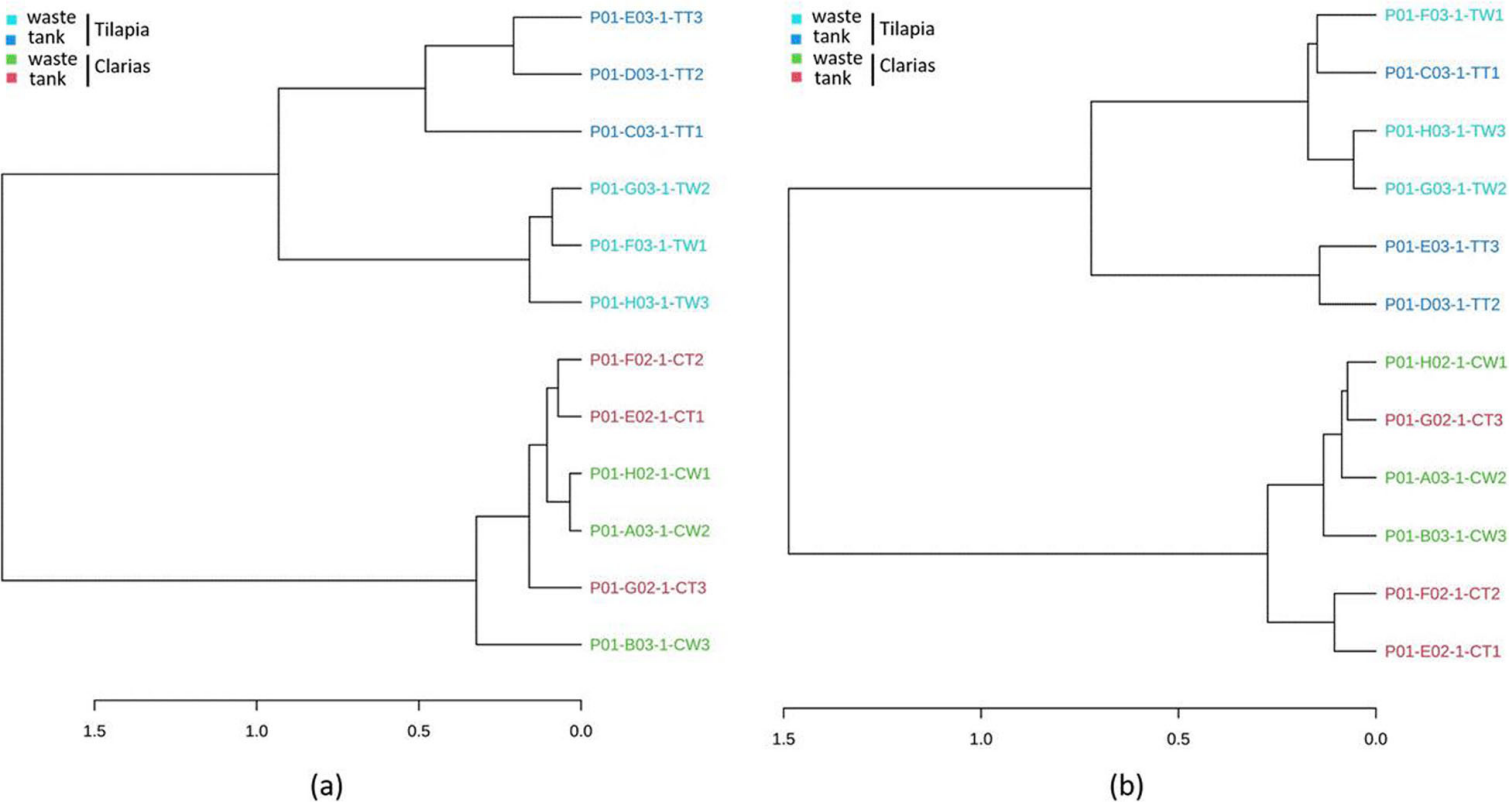
The dendrogram analyses of (**a**) bacterial and (**b**) fungal communities in samples collected from the RAS populated with either Tilapia or Clarias. The samples were collected from the Tilapia water tank (TT), Tilapia waste (TW), Clarias water tank (CT), and Clarias waste (CW)

Dendrogram analyses demonstrated that the bacterial and fungal communities also differed between the Tilapia and Claria cultivation systems (Fig. 5), *inter alia* in the bacterial communities in the water tank and wastewater of the Tilapia RAS (Fig. 5a). However, samples of these communities also shared similarities in their bacterial communities. Fungal communities in Tilapia water tank and waste samples also differed (Fig. 5b). By contrast, there was no clear difference between samples of the Clarias RAS in terms of either bacterial or fungal communities.

**Fig. 6:**
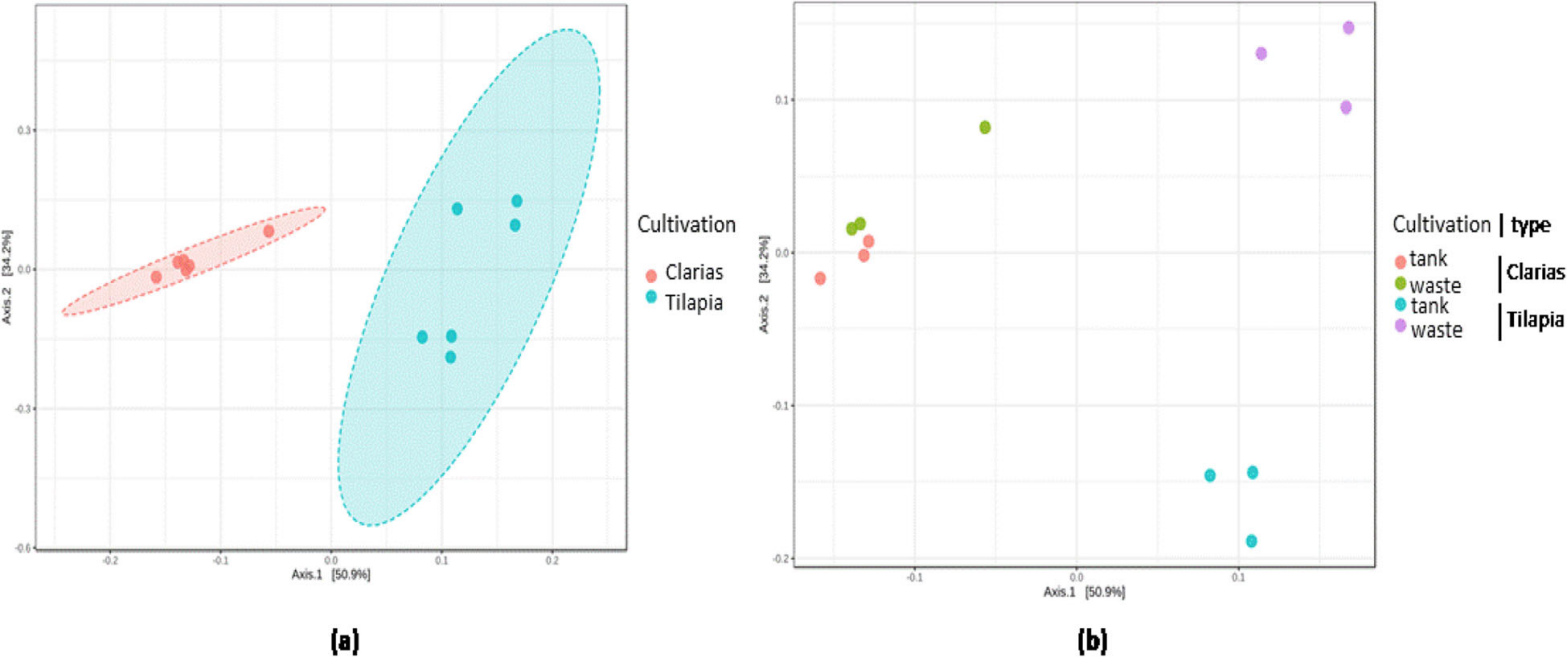
PCoA plots on beta diversity of the bacterial communities distinguished by (**a**) the cultivation system (ANOSIM; *R* = 0.92 and *p* < 0.003) or (**b**) sampling site (ANOSIM; *R* = 0.81 and *p* < 0.001) in the system using Tilapia or Clarias as fish species

**Fig. 7:**
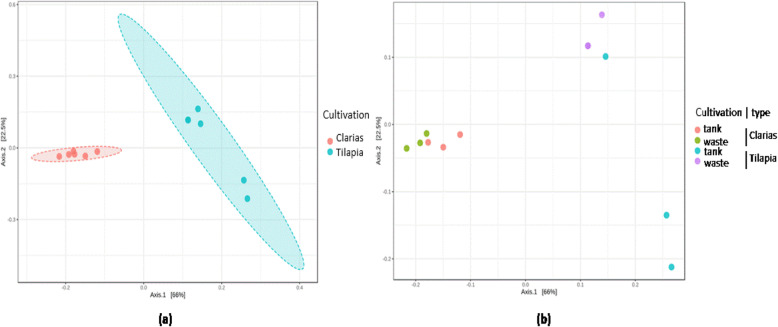
PCoA plots on beta diversity of the fungal communities distinguished by (**a**) the cultivation system (ANOSIM; *R* = 0.05 and *p* < 0.003) or (**b**) sampling site (ANOSIM; *R* = 0.81 and *p* < 0.001) in the system using Tilapia or Clarias as fish species

The beta diversity metrics clearly distinguished the bacterial (Fig. 6) and fungal (Fig. 7) communities associated with the Tilapia and Clarias RAS, and sampling sites in the two systems. The RAS with the two fish species were clearly separated along the first axis of the generated Principal Coordinate Analysis (PCoA) plot (Fig. 6a) and sampling sites (more prominently for samples from the Tilapia RAS than the Clarias RAS) along the second axis (Fig. 6b). No significant differences between sampling site in this respect in the Clarias system were detected. Similar patterns in diversity of fungal communities were also detected (Fig. 7).

**Fig. 8:**
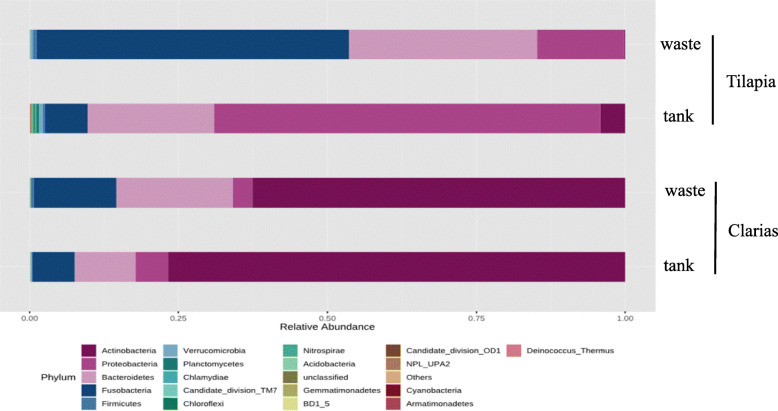
Relative abundance in (%) of dominant bacterial communities at phylum level in water tank, or fish wastes samples collected from RAS populated with Tilapia or Clarias

Analyses of the relative abundance of the bacterial communities showed that the phylum Fusobacteria dominated in samples of Tilapia RAS wastewater, followed by Bacteroidetes and Proteobacteria (accounting for 50, 30 and 20 % of total bacterial OTUs, respectively) (Fig. 8). Tilapia water tank samples were dominated by the phylum Proteobacteria (60 %) followed by Bacteroidetes (20 %), Fusobacteria and Actinobacteria (10 %). Actinobacteria and Proteobacteria dominated in samples of the Clarias RAS. Actinobacteria were more dominant in the water tank (75 %) than the waste samples (60 %). In the Clarias RAS, Bacteroidetes and Fusobacteria were more dominant in the wastewater than in water tank samples. However, in the Tilapia system Actinobacteria were either less abundant than in the Clarias RAS, or completely absent.

**Fig. 9:**
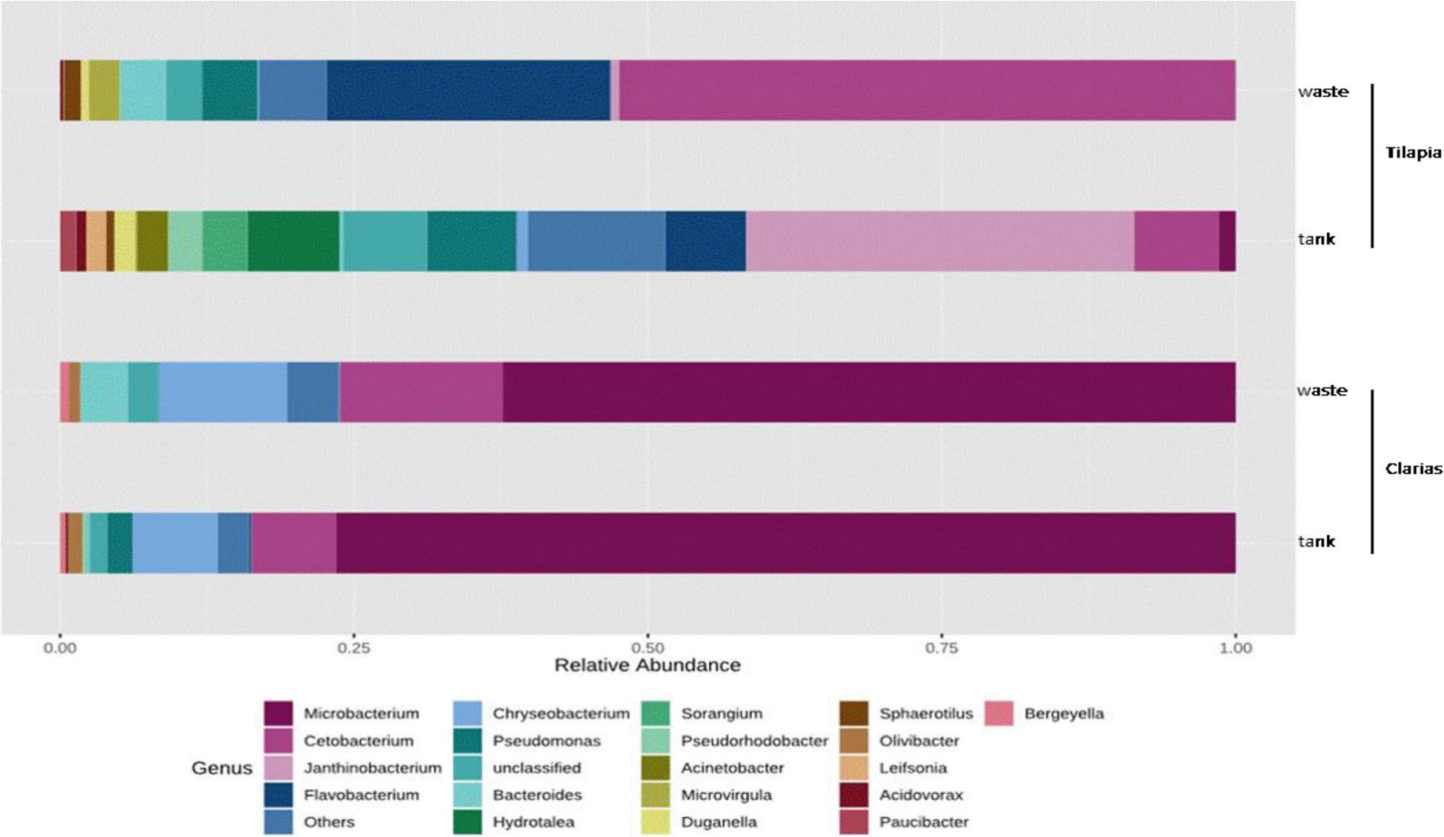
Relative abundance (%) of dominant bacterial communities at the genus level in water tank, or fish waste samples collected from RAS populated with either Tilapia or Clarias as fish species

The bacterial genera *Microbacterium*, *Cetobacterium* and *Chrysobacterium* dominated in samples from the Clarias system (Fig. 9). *Microbacterium* was the most abundant genus in both water tank and waste samples. Tilapia waste samples were dominated by the genera *Cetobacterium* and *Flavobacterium*, followed by *Chryseobacterium*, *Pseudomonas* and *Bacteroides.* Samples from the Tilapia RAS water tank included more genera and were dominated by *Janthinobacterium*, *Flavobacterium*, *Chryseobacterium*, *Pseudomonas*, *Pseudorhodobacter*, *Bacteroides*, *Sorangium* and *Hydrotalea*.

**Fig. 10:**
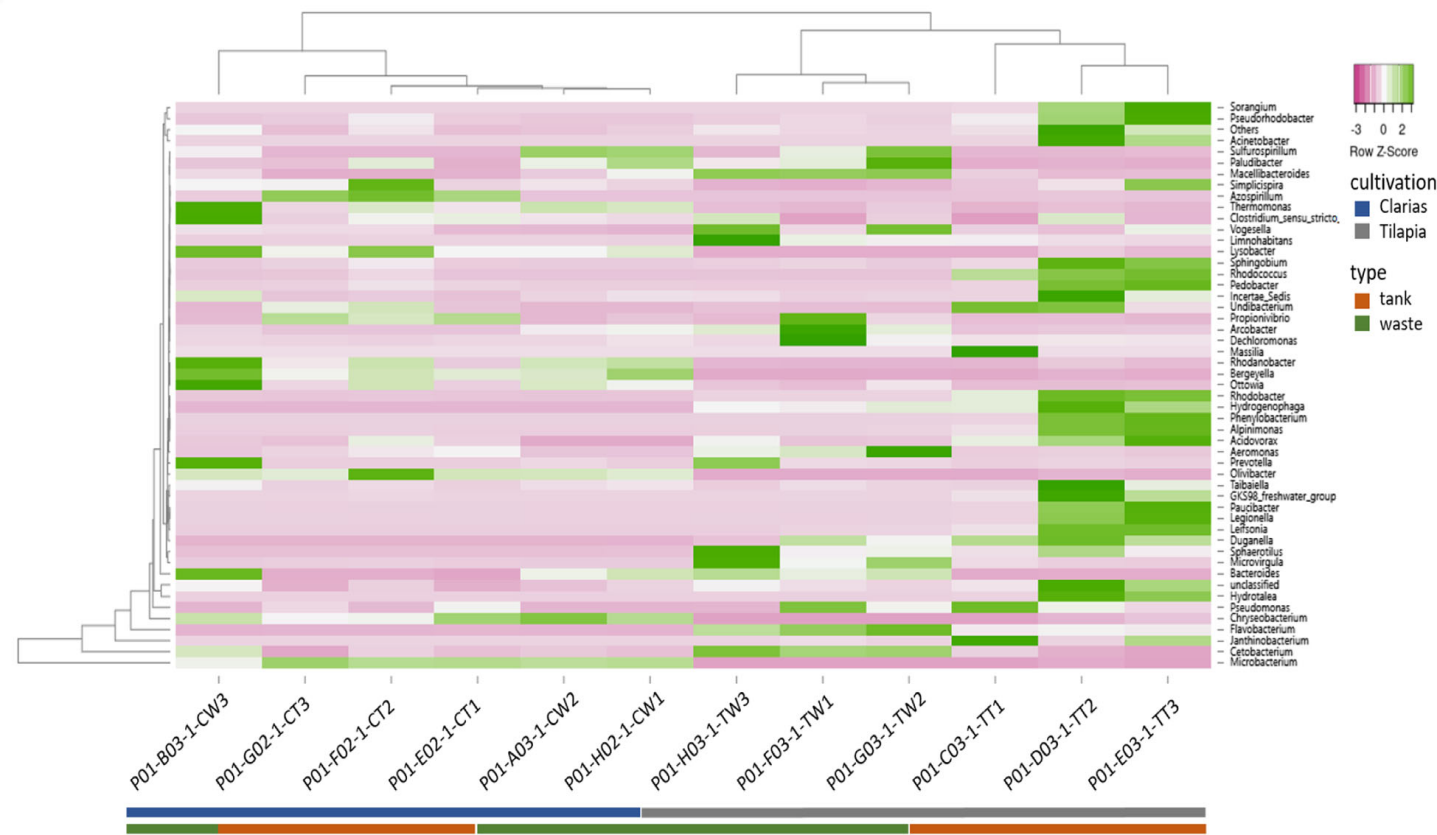
The heatmap shows the abundance of dominant bacterial communities at the genus level in water tank and fish waste samples collected from the Tilapia water tank (TT), Tilapia waste (TW), Clarias water tank (CT), and Clarias waste (CW)

As indicated by the heatmap in Fig. 10, the genera *Sorangium*, *Pseudomonas*, *Pseudorhodobacter*, *Acinetobacter*, *Simplicispira*, *Rhodococcus*, *Pedobacter*, *Rhodobacter*, *Undibacterium*, *Hydrogenobacter*, *Phenyloacterium*, *Alpinimonas*, *Acidovorax*, *Paucibacter*, *Legionella*, *Leisona*, *Duganella* were notably present in Tilapia RAS tank water. Wastewater in this system included the genera *Janthinobacterium*, *Cetobacterium*, *Pseudomonas*, *Aremonas*, *Arcobacterium*, *Clostridium*, *Paludibacter*, *Sulfurospirium*, *Dechloromonas*, *Arcobacter*, *Propionivibrio*, *Limnohabitans*, *Paludibacter* and *Macellibacteroides*. The genera *Chryseobacterium*, *Cetobacterium*, *Simplicispiria*, *Thermomonas*, *Rhodanobacter*, *Ottowia*, *Bergeyella*, *Prevotella* and *Clostridium* were present in Clarias RAS wastewater. The genera *Chryseobacterium*, *Microbacterium*, *Macellibacteroides* and *Azospirillum* were predominant in Clarias water tank samples.

**Fig. 11:**
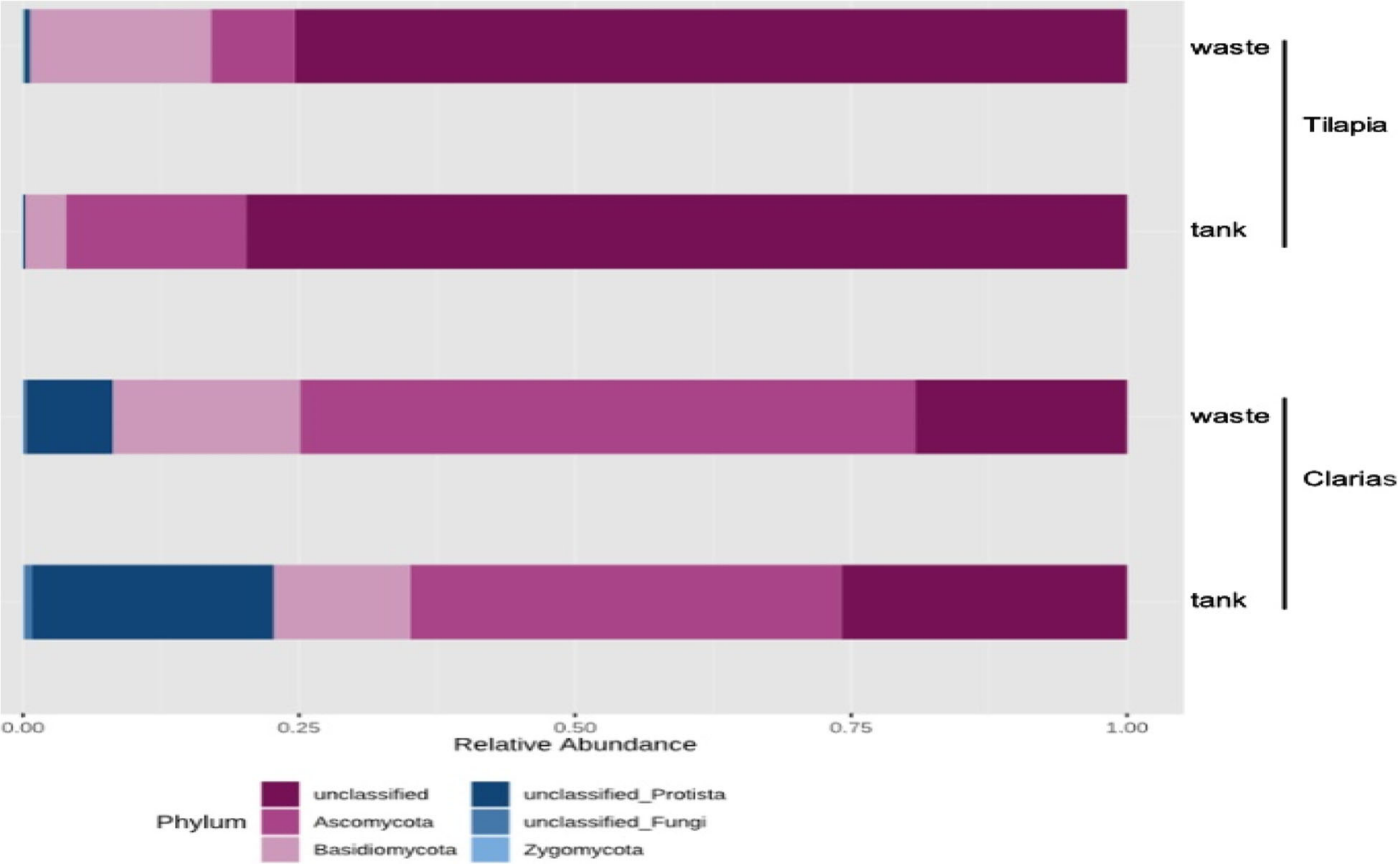
Relative abundance (%) of dominant fungal communities at phylum level in samples collected from water tank and fish waste sites in RAS populated with Tilapia or Clarias

Analyses of the relative abundance of the fungal communities also revealed differences in the dominant taxa depending on the fish species and sampling site (Fig. 11). Most (> 70 %) of the fungal OTUs were unclassified in samples from the Tilapia cultivation system (either water tank or wastewater). However, the phylum Ascomycota was more abundant in samples from the Tilapia water tanks than in samples of Tilapia RAS wastewater, in which the phylum Basidiomycota was more abundant.

There were fewer unclassified fungal OTUs in samples from Clarias RAS water tanks and wastewater than in the Tilapia RAS samples. However, their relative abundances differed between Clarias water tank and waste samples. The waste samples were highly dominated by the phyla Ascomycota, Basidiomycota and unclassified phyla of the kingdom Protista. Water samples from the Clarias system contained Basidiomycota and Ascomycota, but were dominated by an unclassified member of the kingdom Protista (Fig. 11).

**Fig. 12:**
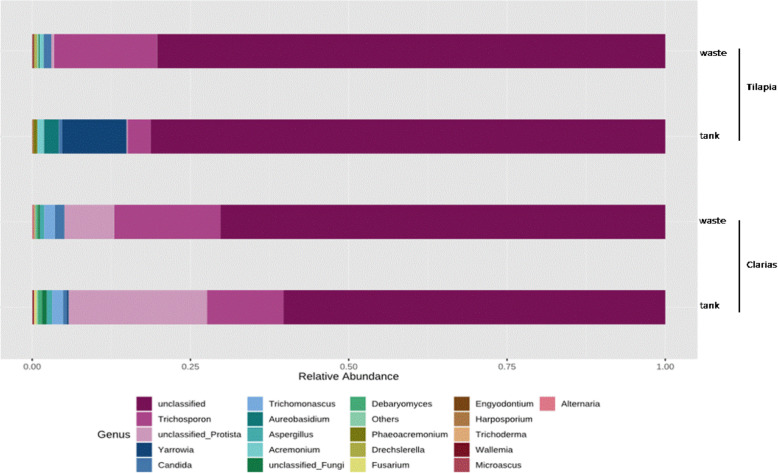
Relative abundance (%) of dominant fungal communities at the genus level in samples collected from water tank and fish wastes in RAS populated with Tilapia or Clarias

Unclassified fungi also dominated at the genus level (70 %) in Tilapia and Clarias RAS (Fig. 12). However, *Trichosporon* spp. were abundant in Clarias RAS tank and wastewater (accounting for 25 % of the total fungal OTUs), and Tilapia RAS wastewater. The genus *Yarrowia* was exclusively found in a Tilapia water tank, while the genus *Trichomonascus* was only detected in the Clarias system.

**Fig. 13:**
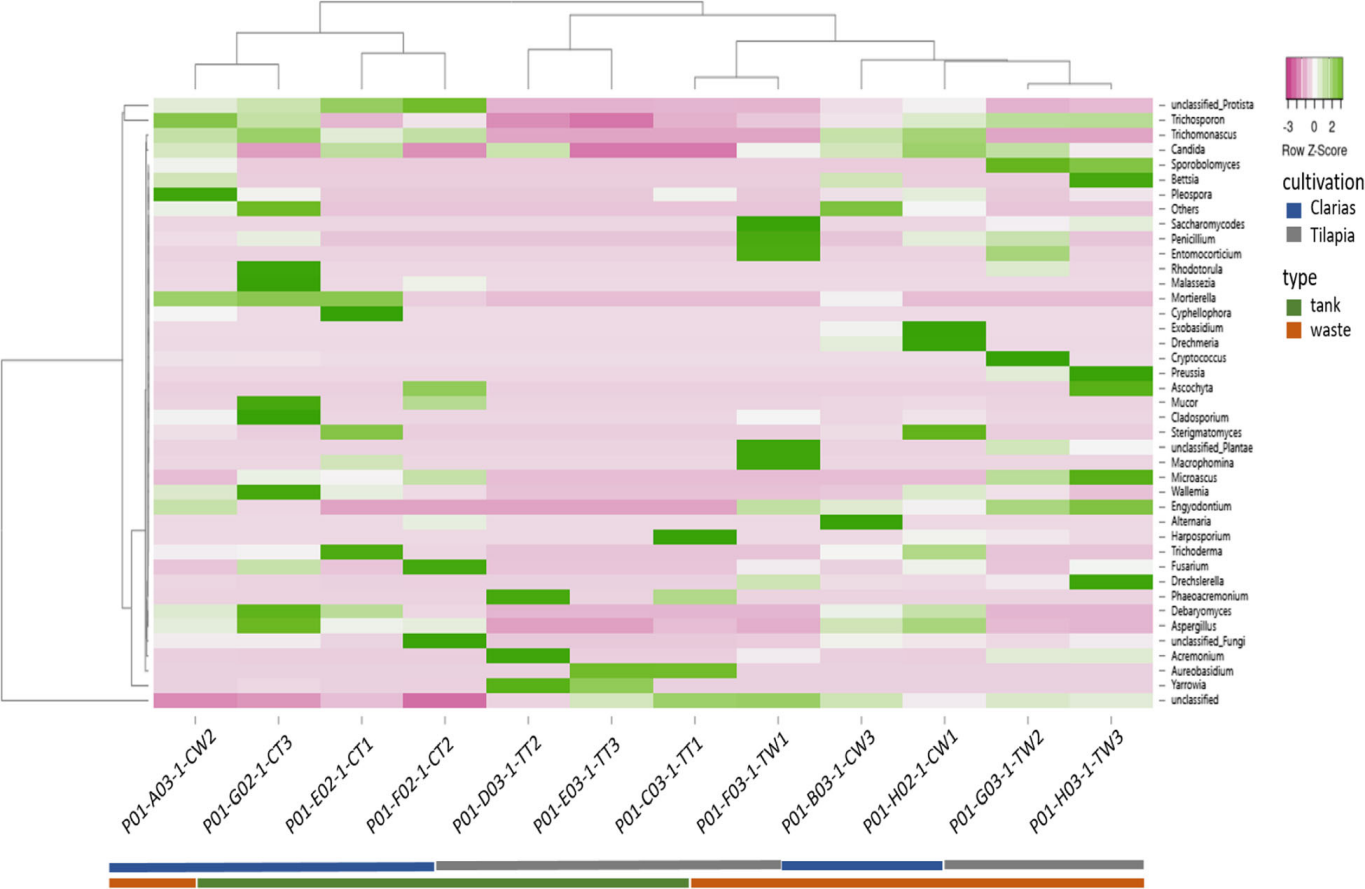
The heatmap presents the abundance of dominant fungal communities at the species level in samples collected from water tanks and fish wastes in RAS populated with Tilapia or Clarias

The fungal communities’ heatmap showed that members of the phylum Basidiomycota in Clarias water tank samples included the genera *Trichosporon*, *Trichomanoascus*, *Phodotonula*, *Malassezia*, *Mortierella*, *Ascochyta*, *Mucor*, *Cladosporium*, *Wallemia*, *Trichoderma*, *Fusarium* and *Deharyomyces* (Fig. 13). The genera *Aspergillus*, *Debaryomyces*, *Wallemia*, *Sterigmatomyces, Exobasidium*, *Trichosporon* and *Pleospora* were found in Clarias RAS waste water. The Tilapia RAS waste water contained more genera than Tilapia water tank samples and was dominated by the genera *Candida*, *Trichosporon*, *Bettsia*, *Sporobolomyces*, *Penicillium*, *Entomocariticium*, *Cryptococcus*, *Preussia*, *Macrophomina*, *Sterigmatomyces*, *Microascuss* and *Enyodonium*. Dominant genera in the Tilapia water tank samples included *Yarrowia*, *Acremonium*, *Harposporium*, *Aureobasidium* and *Phaeoacremonium*.

### Identification of pure cultures and their enzyme activity potential

In total, nine isolates were collected and identified as described in Table 2. *Pseudomonas fluorescens* was the most abundant isolate identified in Clarias RAS wastewater, and both Tilapia RAS water tanks and wastewater. *Pseudomonas veronii* was identified in Tilapia RAS wastewater, and both *Pseudomonas veronii* and *Variovorax paradoxus* strains in Tilapia RAS wastewater.

**Table 2:** Identification and enzyme activities of the pure bacterial isolates isolated from samples collected from water tanks and wastes in RAS populated with Tilapia or Clarias. (+) indicates positive and (-) indicates negative enzyme activities

Number of the isolate	Identification	Collection site	Amylase activity	Protease activity	Phosphatase activity	Cellulase activity	Siderophore production
1	*Pseudomonas fluorescens*	Tilapia water tank	**+**	**+**	+	**-**	**+**
2	*Pseudomonas fluorescens*	Tilapia water tank	**-**	**+**	+	**-**	**-**
3	*Pseudomonas fluorescens*	Tilapia wastes	**+**	**-**	+	**+**	**-**
4	*Pseudomonas veronii*	Tilapia wastes	**+**	**-**	**+**	**+**	**+**
5	*Variovorax paradoxus*	Tilapia wastes	**+**	**-**	**-**	**-**	**-**
6	*Pseudomonas fluorescens*	Clarias water tank	**+**	**+**	**+**	**+**	**+**
7	*Pseudomonas fluorescens*	Clarias wastes	**-**	**+**	**+**	**+**	**+**
8	*Pseudomonas fluorescens*	Clarias wastes	**-**	**+**	**+**	**-**	**+**
9	*Variovorax* spp.	Clarias wastes	**-**	**-**	**-**	**-**	**-**

Regarding enzyme activities, some of the strains isolated from both Tilapias and Clarias RAS waste samples clearly released cellulases (Table 2). Bacterial strains identified in the Clarias system’s wastewater also produced proteases and phosphatases. All the strains isolated from Tilapia RAS wastewater had amylase activities, but not all of them had protease and phosphatase activities. Strains isolated from Tilapia tanks clearly released proteases and phosphatases and one showed amylase activity. Further, most of the strains isolated from Clarias RAS wastewater, and some isolated from Tilapia RAS (water tank and waste) samples clearly released siderophores.

### ***In vitro*** antagonistic activity of bacterial isolates against plant pathogens

**Fig. 14:**
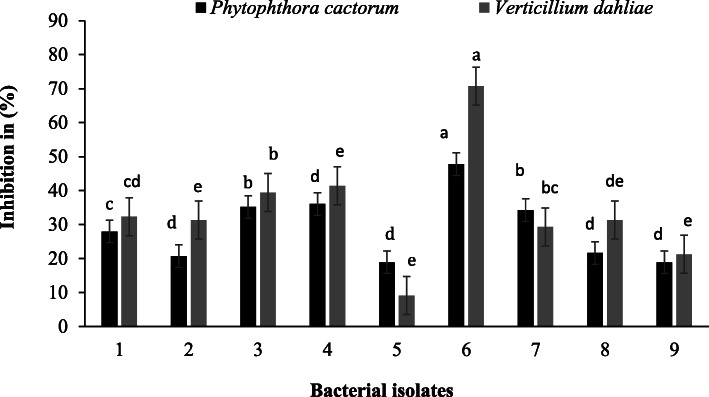
Inhibition (%) of radial growth of the plant pathogens *Phytophthora cactorum* and *Verticillium dahliae* by the bacterial isolates 1–2 from Tilapia water tanks, isolates 3–5 from Tilapia wastes, isolates 6 from Clarias water tanks and isolates 7–9 from Clarias wastes

*In vitro* tests clearly showed that some of the isolated strains were antagonistic towards the plant pathogens *Phytophthora cactorum* and *Verticillium dahliae* (Fig. 14). Isolate 6 (*Pseudomonas fluorescens*) from Clarias water tank samples showed the highest antagonistic potential against both *P. cactorum* and *V. dahliae*. However, *P. fluorescens* (Isolate 6) had higher activity against *V. dahliae* than *P. cactorum*. Apart from *P. fluorescens* Isolate 6, Isolates 3 (*P. fluorescens*) and 4 (*Pseudomonas veronii*) from Tilapia RAS waste had the highest antagonistic activities towards *P. cactorum* and *V. dahliae*. Isolates 2 (*Pseudomonas fluorescens*) from Tilapia water tank and 8 (*Pseudomonas fluorescens*) from Clarias RAS wastewater had higher antagonistic activities towards *V. dahliae* than *P. cactorum*. By contrast, Isolate 5 (*Variovorax paradoxus*) from Tilapia RAS wastewater was more antagonistic towards *P. cactorum* than *V. dahliae*.

### Detached leaf assays

**Fig. 15:**
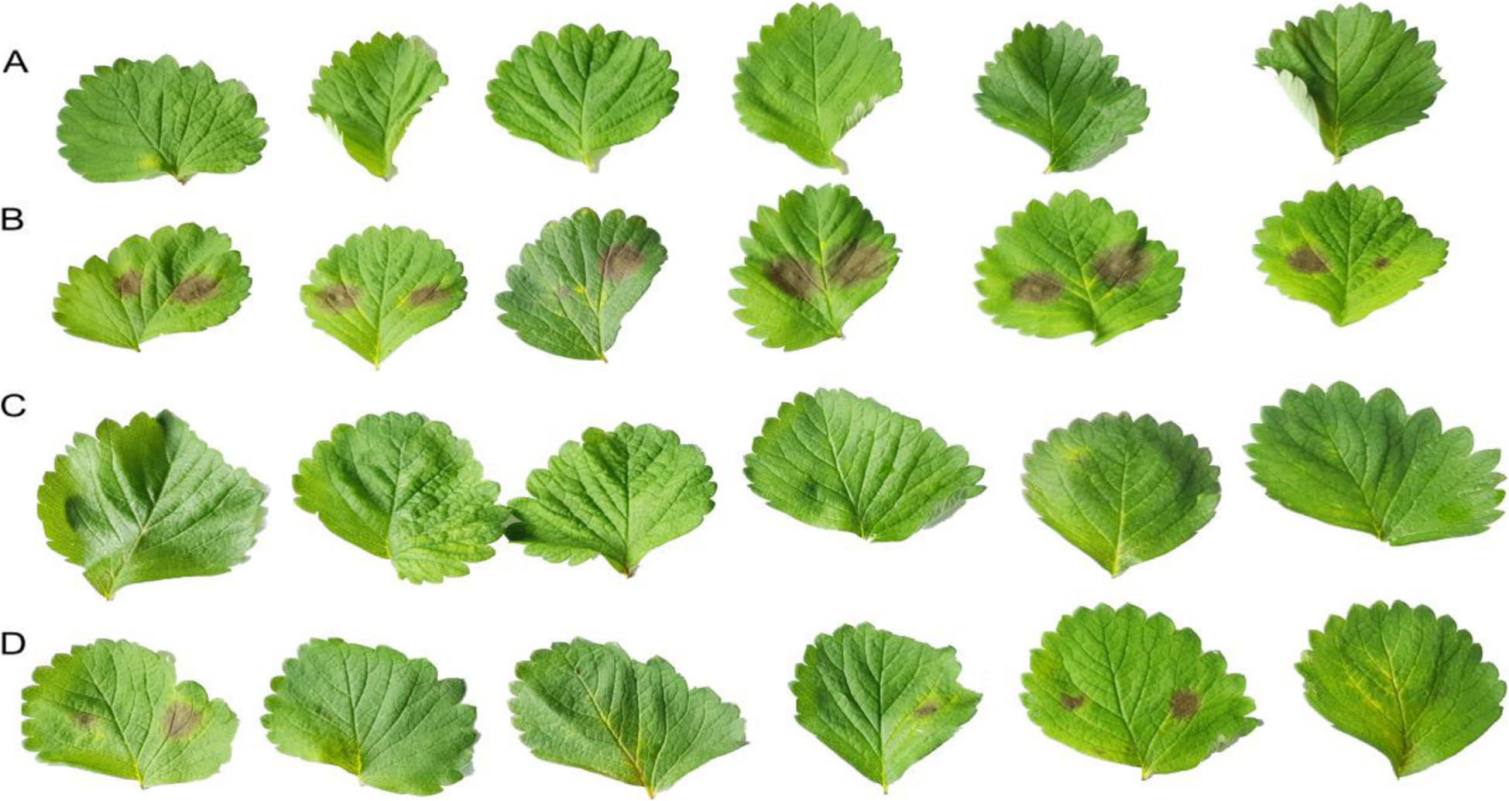
Infection of detached leaflets of strawberry cv. Sonata by *Phytophthora cactorum* isolate RV4 with and without potential antagonistic bacterial isolate*s.* Treatments from top to bottom rows: **A**: water only; **B**: *P. cactorum*; **C**: *P. cactorum* combined with bacterial isolate *Pseudomonas fluorescens* (number 6 from Clarias wastes); **D**: *P. cactorum* combined with bacterial isolate *Pseudomonas veronii* (number 4 isolated from Tilapia wastes). Wild type RV4 formed large disease lesions on treatment B. Treatment C, with isolate number 6, and treatment D with isolate number 4 exhibited no or minimal disease. Leaves were photographed four days after inoculation

Two of the best performing bacterial isolates in the pathogen antagonism tests, *P. veronii* (Isolate 4) and *P. fluorescens* (Isolate 6), were tested for their potential to control *P. cactorum* in detached leaf assays. For this, strawberry (*Fragaria × ananassa* cv. Sonata) leaves were inoculated with *P. cactorum*, and started showing symptoms of infection four days later, while controls inoculated with sterile distilled water remained symptomless. These results confirmed the viability of the zoospore suspensions and absence of the pathogen on uninoculated leaflets. Furthermore, treatment with Isolate 6 (*P. fluorescens*) together with *P. cactorum* showed potent disease inhibition. However, Isolate 4 (*P. veronii*) was less effective for controlling the progression of disease caused by *P. cactorum* (Fig. 15).

## Discussion

Our study provides novel insights into the diversity of microbial communities in commercial RAS, their beneficial effects on plants, and antagonistic potential against plant pathogens. We found that microbial diversity varied depending on the fish species populating the system and sampling point.

The effect of fish species on microbial composition is supported by the beta diversity analyses, which revealed significant differences in the bacterial (Fig. 6) and fungal (Fig. 7) communities of the Tilapia and Clarias RAS.

The fish species in the system had significant effects in the water tank environment, in which the bacterial community was richer in the Tilapia RAS (Fig. 2), but the fungal community was richer in the Clarias RAS (Fig. 4). These findings are consistent with previous indications that the composition of microbial communities is strongly influenced by associated species of fish [25, 26]. The results also agree with previous observations that fungal diversity was higher in a Clarias system than in a Tilapia system [26].

In both Tilapia and Clarias systems, sampling site had no significant effect on the evenness and richness of either bacterial communities (Fig. 2) or fungal communities (Fig. 4). By contrast, the site effect was evident in the composition of the bacterial (Fig. 6) and the fungal (Fig. 7) communities in the Tilapia RAS. Moreover, the dendrogram analyses (Fig. 5) showed there were clear distinctions between the bacterial and fungal communities associated with the two fish species. Differences associated with sampling sites in the Tilapia RAS were also detected. Microbial diversity in RAS is also a function of the water’s physicochemical properties (such as pH and temperature), the feed, gut microbiome of the fish and nutrient contents [27–29]. In this study the cultivation conditions for both fish species were very similar. However, there were substantial differences in their population density and fish biomass per tank was much lower in the Tilapia RAS than in the Clarias RAS (Table 1). This may have considerably influenced unmeasured variables in the water tanks. Previous studies have indicated that the fish gut microbiome can strongly influence the wastewater microbiome [27], but we detected no such indications as microbial diversity in the water tank and wastewater were very similar in both RAS.

The microbial communities of the Tilapia RAS were dominated by bacteria, including the phyla Fusobacteria, Proteobacteria and Bacteroidetes (Fig. 8). Relative abundances of these phyla in the water tank and waste environment differed, primarily due to the dominance of Proteobacteria in the former and Fusobacteria in the latter (Fig. 8). The identified phyla are common in freshwater systems and have been previously detected in Tilapia systems [27]. Our results are also in line with previous findings that Proteobacteria dominated in biofilms in an aquaculture system [30]. Proteobacteria are characterized as r-strategists with an important function in nutrient recycling [31]. Their higher abundance in the Tilapia water tank may have been due to the nutrient conditions in that environment. The high dominance of Fusobacteria in the Tilapia RAS wastewater is consistent with previous indications that the abundance of this genus is related to microbiota in the fish gut, where nutrient breakdown occurs [27]. Our results show that Actinobacteria was the most abundant phylum in the RAS environment with Clarias (Fig. 8). Actinobacteria have received much recent attention for their potential role as probiotic bacteria in marine and freshwater aquaculture [32, 33], further highlighting the importance of the antimicrobial activities of these beneficial bacteria for fish and plant health.

Furthermore, microbial communities in RAS (with and without an aquaponic connection) and Tilapia rearing tanks have been shown to be rich in bacteria with potential capacity to promote plant growth [22, 34]. Our results are consistent with these findings and highlight the Clarias system’s potential to host, in both the tank water and waste water, bacterial genera with plant-growth promoting traits. These include *Microbacterium* and *Cetobacterium* (Fig. 9), which are common rhizobacteria known to have beneficial effects on plant growth [22]. The Tilapia system in our study was richer than the Clarias system in the genera *Pseudomonas*, *Cetobacterium*, *Flavobacterium*, *Sorangium*, *Pseudorhodobacter* and *Bacteroides*, which play a prominent role in promoting plant growth and protecting plants against root pathogens [22]. In addition, the Tilapia RAS tank water (Fig. 10) hosted important genera in biodegradation and alleviation of abiotic stress (which are highly relevant in aquaponic cultivation systems), such as *Simplicispira*, *Rhodobacter* and *Acidovorax* [35, 36]. The identified genera in Tilapia RAS wastewater (Fig. 10) indicate a shift in bacterial composition between the water tank and waste sampling sites. Genera such as *Clostridium*, *Chryseobacterium*, *Janthinobacterium*, *Cetobacterium* and *Pseudomonas* identified in the waste environment also have plant growth-promoting characters [22]. Moreover, the genera *Propionivibrio*, *Sulfurospirium* and *Dechloromonas* are involved in nutrient breakdown, and the removal of certain compounds, e.g., phosphorous by *Propionivibrio* [37]. Samples from the waste site also included genera involved in biodegradation and alleviation of abiotic stress such as *Limnohabitans* and *Paludibacter* [38]. However, the occurrence of *Legionella* and *Aremonas* in Tilapia RAS water tank and wastewater samples requires further investigation as these genera are opportunistic pathogens in aquatic environments and of concern in terms of food safety and human health [39]. On a more positive note, *Pseudomonas fluorescens* has confirmed efficiency for controlling the pathogen *Aremonas* [24], and *Pseudomonas* spp. were detected in both Tilapia and Clarias RAS (Fig. 3). *Pseudomonas fluorescens* is a rhizobacterium of the genus *Pseudomonas*, which we identified in samples from both our RAS (Table 2). Optimizing conditions to favor beneficial microbes in a RAS and the root environment in aquaponic systems might thus be an effective strategy to control fish diseases caused by pathogens in aquaponic systems.

Regarding the fungal communities in our sampled RAS, we found that they included genera with known importance as promoters of plant growth and antagonists towards plant pathogens. These genera included: *Cladosporium*, *Mortierella* [40] and *Trichoderma* [41] in Clarias RAS tank water: *Pleospora* [42] in Clarias RAS waste water; *Acremonium* [43] in Tilapia RAS tank water and *Penicillium* [40] in Tilapia wastewater samples (Figs. 12 and 13). However, these systems also contained potentially pathogenic genera for humans and plants, such as *Fusarium* [43], *Candida* and *Cryptococcus*, which pose significant challenges that need further investigation. Further investigation of fungal sequences and appropriate primers is also needed to enable identification of fungi that could not be classified beyond the level of phylum or genus in this study.

The bacterial strains isolated from the Tilapia system showed clear potential to inhibit development of symptoms of disease caused by two well-known plant pathogens and hence as biocontrol agents in aquaponic systems. The only isolated bacterial strain that showed such potential and was positively identified and present in all samples was *Pseudomonas fluorescens*. However, log transformation of the *Pseudomonas* data we obtained indicates that the other strains were also present in all the investigated samples (Fig. 3), thus strengthening our findings concerning the isolated strains. Other less ubiquitous taxa were *Pseudomonas veronii* and *Vaiovorax paradoxus* in Tilapia RAS wastewater, and *Variovoras* spp. in Clarias RAS wastewater (Table 2). *Pseudomonas* spp. generally are important microorganisms that can promote plant growth as well as producing antimicrobial substances that control plant pathogens [18, 20]. *Pseudomonas veronii* specifically has high bioremediation potential and is found in both soil and water environments [44]. *Vaiovorax paradoxus* and other *Variovoras* species also have known ability to promote plant growth [45].

Enzyme production is one of the modes of action through which microorganisms combat pathogen attack [20]. In aquaculture, probiotic microbial taxa such as Actinobacteria, Proteobacteria and Bacteroidetes release various enzymes, such as proteases, chitinases, glucanases, amylases, cellulases, and phosphatases that break down corresponding nutritional components in their substrates [46]. Results of this study indicate that most of the strains isolated from the Tilapia system produce proteases, phosphatases, cellulases and siderophores (Table 2). *Pseudomonas veronii* isolated from Tilapia RAS wastewater (Isolate 4) exhibited ability to produce all of these enzymes. *Pseudomonas fluorescens* isolates from Clarias RAS tank water (Isolate 6) and Tilapia RAS wastewater (Isolate 3) could also produce most of the enzymes. *Pseudomonas* spp. isolated from both RAS systems are well-known for producing siderophores in plant roots that improve the availability of iron by aiding its uptake by plant roots [47]. Thus, the isolates in our study may be good candidates for use in aquaponic systems to increase the availability of phosphorous and iron for plant growth.

*In vitro* investigations of the antagonistic abilities of the isolated strains highlighted differences in their inhibitory effects on the growth of pathogens. *Pseudomonas fluorescens* (Isolate 6), *Pseudomonas fluorescens* (Isolate 3) and *Pseudomonas veronii* (Isolate 4) suppressed growth of the root pathogen *Phytophthora cactorum* more than the other strains (Fig. 14). This difference may be due to differences in cellulase production by these strains, which not only breaks down a component in the feed, but can also break down cell walls of pathogens such as *P. cactorum*. However, *in vivo* antagonistic effects are needed in aquaponics for these results to have practical applicability. Our detached leaf assay provides strong evidence of the antagonistic potential of *P. veronii* (Isolate 4) and *P. fluorescens* (Isolate 6) against *P. cactorum* (Fig. 15).

## Conclusions

The current study has contributed new knowledge concerning the role of microbial tools in commercially based recirculated aquaculture systems (RAS) as promoters of plant growth and suppressors of disease. This knowledge strengthens the potential application of RAS as a part of aquaponic systems, which currently face challenges regarding plant nutrients and pathogens. The assemblages of microbial taxa at the level of phyla and genera both in Tilapia and Clarias RAS suggest the suitability of these systems to be used in aquaponic cultivation. However, in terms of promoting plant growth, plant protection and biodegradation, the characteristics of the richness and composition of the microbial communities in the Tilapia system make it the better choice for application in aquaponic systems. The commercial Clarias RAS could also be applied in aquaponic systems, but principally from a probiotics perspective due to the dominance of Actinobacteria in this system. *Pseudomonas* spp. from both Tilapia tank water and waste samples, and Clarias wastes, are good candidates with the potential to produce extracellular enzymes that enhance nutrient uptake. Although the results suggest considerable potential for using microbial communities to manage and control certain aspects of aquaponic systems, our findings need to be strengthened with *in vivo* studies to explore further the inhibition of plant pathogens and positive effects on plant nutrition. Risks arising from the presence of pathogens also need further investigation. However, our results can still be used as a foundation for the design of aquaponic systems populated with either Tilapia or Clarias. Still, these results need further investigations considering the microbial patterns in the RAS of Tilapia and Clarias in relation to the abiotic and biotic factors in the system.

## Materials & Methods

### Sample collection

Two commercial warm and fresh water RAS populated with Tilapia (*Oreochromis niloticus*) or Clarias (*Clarias gariepinus*) as the fish species and no aquaponic connection were used as the experimental units. The RAS of Tilapia consisted of a water tank of 70 m^3^ and of 150 m^3^ of the Clarias system. A filtration unite is connected the system including a big biofilter and a Degas column for the removal of nitrogen and carbon dioxide and addition of oxygen to the system. Three independent replicates (each 5 L water samples) were collected at two different sites, viz. from the fish water tanks and from the wastewater where the fish faeces were accumulated. Both RAS with each respective fish type were similarly sampled. The collection site was considered as a treatment. Thus, in total, three fish water samples (biological replicates) and three wastewater samples (biological replicates) were randomly collected from the Tilapia and Clarias systems respectively. The samples were then transferred to the laboratory for subsequent microbial analyses.

### Viable count and microbial enumeration

The viable count method was used to quantify the microbiota in each treatment. Dilution series and enumeration on selective agar media were applied [48]. From this dilution stock, 200 µL aliquots were spread, in triplicate, on the following media: (i) 0.1 % Tryptic soya agar (TSA, DIFCO 0369-17-6) complemented with cycloheximide (100 µL mL-1) to enumerate the general bacterial flora; (ii) 0.5 % malt extract agar (MA, DIFCO 0186-17-7) to enumerate the general fungal flora; and (iii) King Agar B (KB) with cycloheximide (100 µg mL-1) to enumerate the fluorescent pseudomonads. The MA plates were incubated at room temperature for seven days and the TSA and KB plates were incubated for 24 h at 25 °C.

### Microbial community analyses

#### Samples preparation

The total microbiome analyses started by sterile filtration of 1 L of the collected samples through 0.2 μm filters using bottle-top vacuum filtration systems, PES (WVR- Sweden, 514 − 0332). The filtration unit filter was then transferred to a 50 mL tube, washed with 50 mL sterile autoclaved water, followed by vigorous vortexing for 2 min. The collected material was then centrifuged at 5000 rpm for 10 min and the pellets were stored at -80 °C.

#### DNA extraction

The DNA extraction was performed using Enzymo DNA preparation kit (D 4300, Sigma Aldrich) following the manufacturer’s recommendations.

#### Illumina sequencing

The bacterial and fungal communities were sequenced with an Illumina MiSeq (2 × 300 bp) at LGC Genomics GmbH (Berlin, Germany) [49] using Illumina bcl2fastq 2.17.1.14 software. The bacterial 16 S ribosomal gene was targeted using the forward primer 341 F (5′-CCTACGGGNGGCWGCAG-3′) and the reverse primer 785R (5′-GACTACHVGGGTATCTAATCC-3′). The fungal forward primer ITS7F (5′-GTG ART CAT CGA ATCTTTG GTT G-3′) and the reverse primer (5′-TCC TCC GCT TAT TGA TAT GC-3′) were used to target the ITS2 region for fungal assessment. Data pre-processing and OTU picking from amplicons were performed using MOTHUR pipelines (version 1.35.1). Reads with a final length of < 100 bases were discarded and primer, barcode sequences as well as chimeras were removed. For taxonomical classification, alignment against 16 S Mothur-Silva SEED r119 reference was performed and sequences from other domains of life were removed. Assignment of operational taxonomic unites, OUTs, was performed at the 97 % identity level using the cluster split method. The fast Tree method was used to generate the phylogenetic trees for 16 S and ITS, respectively.

### Microbial activities

#### Bacterial pure cultures

For each treatment, two single colonies from the TSA plates and two from the KB plates were selected and transferred to be grown on broth media of tryptic soya broth (TSB) and King B broth (KBB), respectively. One loopful of culture was inoculated into 15 mL of the broth media and incubated at 25°C with shaking (140 rpm) for 24 h. Bacterial DNA was extracted using the Quick-DNA Bacterial Microprep Kit according to the manufacturer’s recommendations (Zymo Research, USA). The DNA yield and integrity was assessed using a NanoDrop Micro Photometer (NanoDrop Technologies, UK), and agarose gel electrophoresis, respectively. The 16s rRNA region of all bacterial isolates was PCR amplified individually with the universal primer pairs, 27F (5’- AGAGTTTGATCMTGGCTCAG-3’) and 907R (5’-CCGTCAATTCMTTTRAGTTT-3’) [50]. PCRs were performed using ten ng of DNA with the following temperature parameters: initial denaturation step at 94 °C for 3 min, followed by 35 cycles at 94 °C for 45 s, 50 °C for 30 s, and 72 °C for 30 s, followed by a final extension step of 72 °C for 5 min. The PCR products were purified using the Qiagen QIAquick PCR Purification Kit (Qiagen, UK). Sanger sequencing for species identification was carried out at the GATC biotech AG sequencing facility (Germany) using 27 F and 907R primers. DNA star software was used (DNASTAR, USA) to analyze and edit nucleotide sequences obtained from the sequencing platform manually. Resulting sequences with 16 s region were searched for matching hits against the National Center for Biotechnology Information (NCBI) GenBank non-redundant nucleotide database (BLASTn; [51]). Search hits to sequences from records in the database were evaluated for coverage and identity and the best matched NCBI accession was recorded.

#### Enzyme activities

Functional characters of the isolates were assessed by assaying their enzymatic activities. The isolated bacterial colonies were screened for amylase, protease, phosphatase, siderophores and cellulase production on functional media in plate assays [52]. The M9 Minimal Salts medium (VWR, Sweden) was used as a base medium. For amylase assays, the isolates were inoculated on M9 media amended with 1 % (w/v) starch and incubated for 24–72 h at 20 ± 2 °C. After incubation, the plates were flooded with Lugol iodine, and a zone of clearance around colonies against the resulting dark background was taken as an indication of amylase production.

For assessing protease activity, the isolates were inoculated on M9 plates amended with skimmed milk (20 mL L^− 1^), incubated for 24–72 h at 20 ± 2 °C, and formation of a halo around the colonies indicated protease production. The same incubation conditions and indicative criteria were used for assessing cellulase production. For this, the M9 plates amended with (1 %) carboxymethyl cellulose were used, which were flooded with Congo Red solution (0.2 % w/v) for 30 min then washed with 1 M NaCl solution.

For phosphatase activity assays, tryptose phosphate agar plates supplemented with Methyl Green (0.05 mg mL^− 1^) were used. The plates were incubated for 5 days at 20 ± 2 ^0^ C. Development of green coloration, after incubation, indicated positive phosphatase activity. Siderophores production was also assessed, using Chromazurol S agar plates inoculated with bacterial isolates and incubated for three days at 20 ± 2 ^0^ C. The formation of an orange halo around the colonies indicated siderophores production.

#### In vitro antagonistic assay

The bacterial strains isolated were screened for antagonistic potential towards the oomycete/fungal pathogens *Phytophthora cactorum* and *Verticillium dahlia*. The assay was performed on cornmeal agar (CMA) plate agar plates with the mycelial plug (1 × 1 cm) of the test pathogen placed in the centre followed by streaking test bacterial isolate three cm apart on either side of the pathogen plug and monitored for ten days at 20 °C. Controls constitute only the test pathogens. The experiments were repeated thrice with three replicates for each independent experiments. The radial growth of the pathogen growth towards test bacteria was measured and the Growth Inhibition Percentage (GIP) was calculated according to the following formula:

$$ \left(\%\right)=\left( RC- RT\right)/ RC\ x\ 100. $$

where RC constitutes radial growth of the pathogen in the control plate (cm) and RT is the radial growth of the pathogen (cm) in the treated plate [53].

#### Detached leaf assay (DLA)

The antagonistic potential of bacterial isolates towards the strawberry pathogen *P. cactorum* was tested in a detached leaf assay. The best performing bacterial isolates with the highest antagonistic activity from the *in vitro* studies were selected for the DLA assay. The bacteria were cultured overnight on Tryptic Soy Broth media at 28 °C and pelleted. The pellet was washed and resuspended in distilled water, followed by density measurement and adjustment to OD 0.1 at 600 nm.

For zoospore production, *P. cactorum* isolate RV4 was cultured as described [54]. For sporangia formation, the agar plugs from the outer edges of freshly growing mycelium colonies were subjected to dark conditions on V8 media for three days followed by treatment with autoclaved soil extract solution under light conditions for 48 h. The sporangial suspensions obtained were subjected to cold treatment at 8 °C to release the zoospores over an average 2–3 h period depending on the zoospore’s release efficiency. The zoospore inoculum was collected and counted using a haemocytometer to adjust inoculum to 20 000 zoospores per mL.

Young strawberry leaflets (cv. Sonata) grown in controlled conditions were used to assess disease development. The leaves were placed adaxial side facing up on moist paper in clear plastic containers and inoculated with a mixture of zoospore and bacterial suspension with approximately 20,000 zoospores and OD = 0.1 bacterial suspension in a 20 µl droplet on either side of the leaf.

midrib. The controls constituted either sterile water or pathogen only. The plastic containers were tightly sealed and placed at 20 °C with 16 h photoperiod. Pathogenicity phenotypes were photographed and assessed at 4 days post-inoculation (DPI) using the image-processing program ImageJ. Each independent experiment was evaluated with six replicates and the experiments were repeated thrice.

### Statistical analyses

All statistical analyses were performed within Microbiome Analyst v1.0 [55]. OTUs were pre-filtered before conducting any statistical analysis by retaining those present in at least two samples. The data were normalized using the total sum scaling method (counts per million normalization) to address variability in sampling depth. Alpha diversity profiling was conducted using Shannon and Chao1 diversity indices and statistical significance testing using analysis of variance (ANOVA). To examine differences between the different groups in community compositions, beta diversity was calculated using unweighted Unifrac Distance and statistical comparisons performed with ANalysis of SIMilarities (ANOSIM). Patterns of sample dissimilarity were visualized using Principal Coordinate Analysis (PCoA) plots. In addition, hierarchical clustering and heatmaps were constructed using the Ward clustering algorithm and Euclidean distance measure to examine the robustness of sample clustering and relative abundances. The results from the microbial enumeration were log-transformed to meet the assumptions of homogeneity and normality. The significance effect of treatment was tested by ANOVA followed by Tukey’s multiple comparison test (p < 0.05) using Minitab v16. To explore how top taxa differed between the different cultivation/waste types, classical univariate statistical comparisons were inferred using t-test/ANOVA method with an adjusted p-value cutoff of 0.05.

## Data Availability

The sequenced data were submitted to Genbank database and got the submission ID (SUB8541601) and the bioProject ID (PRJNA702651). The project information are accessible with the following link: https://www.ncbi.nlm.nih.gov/sra/PRJNA702651
